# Gait Recognition Method of Underground Coal Mine Personnel Based on Densely Connected Convolution Network and Stacked Convolutional Autoencoder

**DOI:** 10.3390/e22060695

**Published:** 2020-06-21

**Authors:** Xiaoyang Liu, Jinqiang Liu

**Affiliations:** School of Mechanical Electronic & Information Engineering, China University of Mining & Technology, Beijing 100083, China

**Keywords:** underground coal mine personnel, gait recognition, similarity learning, densely connected convolution network, stacked convolutional autoencoder, Two-Stream neural network

## Abstract

Biological recognition methods often use biological characteristics such as the human face, iris, fingerprint, and palm print; however, such images often become blurred under the limitation of the complex environment of the underground, which leads to low identification rates of underground coal mine personnel. A gait recognition method via similarity learning named Two-Stream neural network (TS-Net) is proposed based on a densely connected convolution network (DenseNet) and stacked convolutional autoencoder (SCAE). The mainstream network based on DenseNet is mainly used to learn the similarity of dynamic deep features containing spatiotemporal information in the gait pattern. The auxiliary stream network based on SCAE is used to learn the similarity of static invariant features containing physiological information. Moreover, a novel feature fusion method is adopted to achieve the fusion and representation of dynamic and static features. The extracted features are robust to angle, clothing, miner hats, waterproof shoes, and carrying conditions. The method was evaluated on the challenging CASIA-B gait dataset and the collected gait dataset of underground coal mine personnel (UCMP-GAIT). Experimental results show that the method is effective and feasible for the gait recognition of underground coal mine personnel. Besides, compared with other gait recognition methods, the recognition accuracy has been significantly improved.

## 1. Introduction

Gait recognition is a new biometric recognition technology that can recognize the identity of people based on walking posture [1]. Gait recognition has a unique advantage over other biometric technologies, namely the recognition potential at long distances or with low video quality. In addition, the gait is difficult to hide or camouflage and does not require people to cooperate with [2]. Especially in the dark, infrared gait recognition technology can play its role. At present, the identification of people in coal mines is mostly based on faces and fingerprints. Although the recognition technology based on faces and fingerprints has matured and has a high recognition rate in normal environments, due to limited space, dim light, moist air, coal dust in the roadway, etc., face and fingerprints are often blurred, seriously affecting the recognition rate of these identification methods [3]. The gait recognition method does not have more requirements for illumination, video quality, or distance, which is very consistent with the environmental characteristics of underground coal mines. By identifying and monitoring the gait images of the personnel, the identity information of the underground operating personnel can be accurately identified the first time. This is of great significance for the realization of mine safety monitoring and personnel identity positioning.

**Figure 1:**
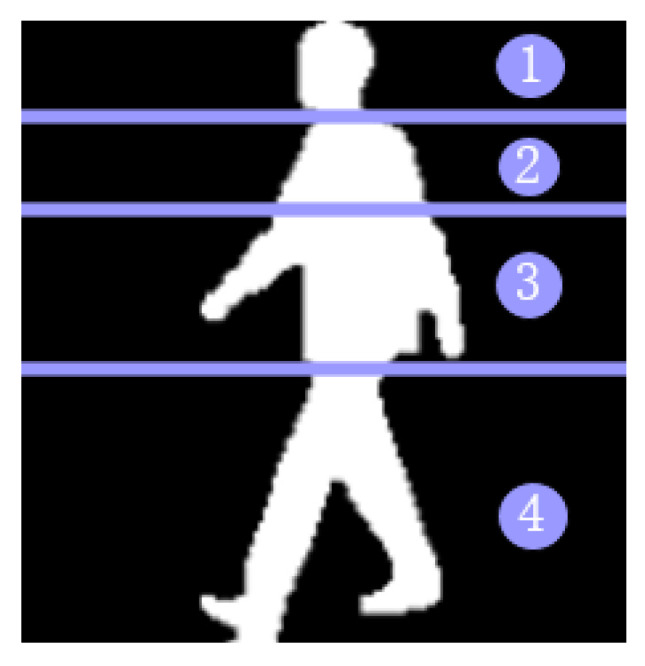
Gait recognition key areas.

Gait recognition is a global recognition mode, but the main concern is gait information. Human body characteristics include static invariant features (physiological characteristics) and dynamic deep features (space–time characteristics). Static invariant features include body size (short and tall, fat and thin), head shape, shoulder width, etc. The dynamic deep features include the amplitude of the step, the frequency of the step, the center of gravity of the body, the coordination of the legs, and the swing amplitude of the arm. If the human body is divided into four parts, for gait recognition, the key areas of recognition are shown in Figure 1. The contribution of each part of the body to recognition is 4 > 1 > 3 > 2.

In [4], the gait features are extracted in the form of interval-valued symbol features using the distance relationship between the minimum inertia axis and the outermost contour. In [5], the author proposed a view-invariant gait recognition method based on Kinect skeleton features. The author uses gait energy image (GEI)-based local multi-scale feature descriptors for human gait recognition in [6]. Reference [7] proposed a gait recognition method based on a static energy map and dynamic group hidden Markov model, which has certain robustness to angle changes and reduces the impact of noise on recognition. In [8], the time–frequency domain features of the gait image were extracted using wavelet transform and time–frequency analysis methods to perform gait recognition. The author proposed a gait recognition method based on tensor discriminant analysis and Gabor feature extraction in [9]. This method is a linear quantification of linear discriminant analysis (LDA). Its advantage is that it does not need to convert gait images into vectors. Therefore, the problem of having a “small sample” is overcome.

With the rise and maturity of deep learning methods such as residual neural network (ResNet) [10] and generative adversarial network (GAN) [11], deep learning has become one of the most popular methods for solving gait recognition. A cross-perspective gait recognition method based on deep convolutional neural network proposed by Huang et al. [12] of the Institute of Automation of the Chinese Academy of Sciences can perform multi-perspective recognition, with improved accuracy. Chen et al. [13] proposed a GaitGAN gait recognition method based on GAN. This method uses GAN to transform gait images at any viewing angle and any state into gait images at a 90° normal walking state, with high recognition accuracy and fast speed. Fudan University proposed a GaitSet algorithm based on a gait contour graph [14]. Regarding gait contours as a set of images without time-series relations, instead of deliberately modeling the time series of gait contours, the study makes deep neural network optimize itself to extract and use this relationship. In [15], the author developed an efficient spatiotemporal gait features with deep learning. The extracted spatial and temporal gait features are embedded into the null space to obtain the similarity of gait image pairs and thereby achieve gait recognition. In [16], the author used CNNs and multiple loss functions to extract gait features from silhouette sequences and GEIs, respectively. This method can better extract spatiotemporal information based on appearance. Wang et al. [17] proposed a DL-based gait recognition method named Two-branch Convolution Neural Network. This method uses two kinds of CNN models to extract gait features, and then trains an SVM classifier with the output of each CNN model to achieve gait recognition. In [18], the author proposed an end-to-end system based on pre-trained DenseNet-201 [19] model for features extraction to realize gait recognition. This method achieved a high recognition rate.

**Figure 2:**
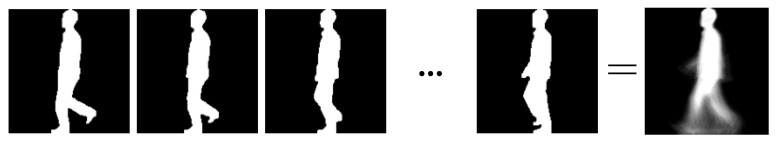
Gait energy image (GEI).

Aiming at the problems that the existing gait recognition methods have low accuracy and cannot simultaneously extract the dynamic and static features of human walking, we propose a Two-Stream neural network model based on similarity learning. [20,21]. Our proposed model takes the gait energy image (GEI) as the model input [22]. GEI contains both human physiological information and spatiotemporal information during walking. The gait image sequence is summed and averaged into one gait picture, as shown in Figure 2.

## 2. Overview of the Proposed TS-Net Model

**Figure 3:**
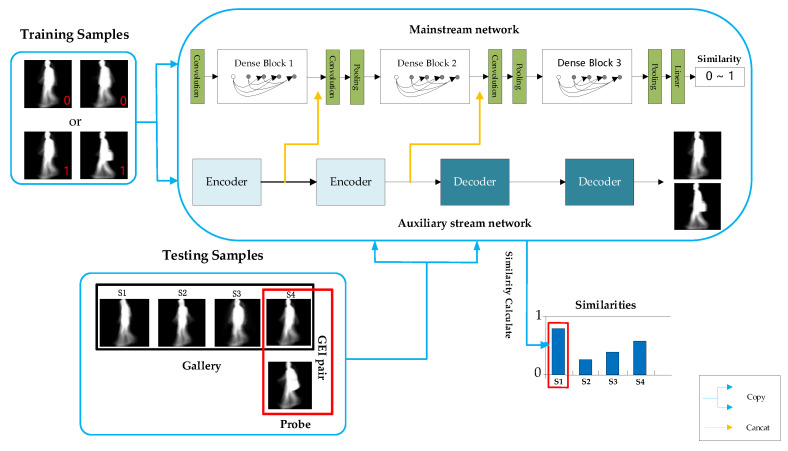
The architecture of the proposed Two-Stream neural network (TS-Net) model.

The proposed Two-Stream neural network (TS-Net) model simultaneously extracts dynamic deep features and static invariant features in gait images, and it fuses multiple resolutions static invariant features with dynamic deep features to obtain the most discriminating spatial–temporal information. In addition, the recognition task is transformed into a binary classification problem through similarity learning methods, which can more accurately achieve personnel recognition. As shown in Figure 3, the TS-Net model proposed in this paper mainly consists of three parts: dynamic and static feature extraction, feature fusion, and recognition.

Dynamic and static feature extraction consists of two parallel networks: the mainstream network based on DenseNet [19] and the auxiliary stream network based on stacked convolutional autoencoder (SCAE) [23,24]. Firstly, in the mainstream network, dynamic deep features are extracted from gait image, which represent more macroscopic and abstract spatiotemporal information of the gait image. In the auxiliary stream network, static invariant features are extracted from gait image samples, which represent physiological information such as the body shape and head shape of the low-dimensional gait image.

In the dynamic and static feature extraction process, the gait features extracted from the auxiliary stream network are integrated into the mainstream network, and the final gait image features are obtained through the mainstream network to learn the gait similarity and then predict whether the input image pair belongs to the same person. Finally, during the test, an image pair consisting of the probe view and the gallery view is input to the network, and the similarity of the image pair is obtained to realize gait recognition. The three parts of the TS-Net model will be described in detail next.

## 3. Dynamic and Static Feature Extraction, Fusion, and Recognition

### 3.1. Mainstream Network

Gait images are high-dimensional, complex, and changeable non-linear data. To extract discriminative spatiotemporal information from gait images, it is necessary to build a deeper network. Studies shows that increasing the number of layers in the network can help extract more hierarchical features, and the deeper the network, the better the expression ability. However, in practical applications, as the number of stacked layers increases, training and convergence will be difficult. Although methods such as batch normalization (BN) can be used to alleviate the vanishing gradient and explosive gradient problems [25,26], the performance of the network will still decline. This degradation is not caused by overfitting, but the network is too deep to make it difficult to train. Therefore, the traditional neural network model cannot build a sufficiently deep network.

**Figure 4:**
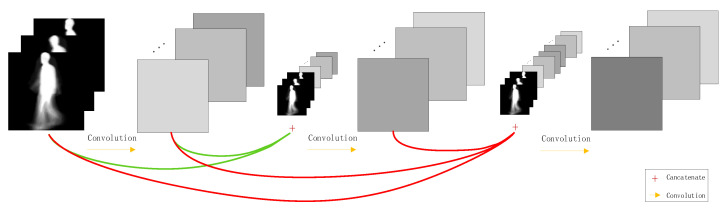
Connections for densely connected convolution networks.

Densely connected convolution network (DenseNet) can build deeper networks. Traditional neural networks have only one connection between each layer and subsequent layers; that is, a convolutional network with L layers has L connections. Compared with the traditional neural network, in order to improve the information flow between layers, the DenseNet is connected to each layer in a feed-forward manner, and each layer has a direct connection with the subsequent layers [27,28,29]. The network will have L(L+1)/2 dense connections [19]. For each layer, the inputs of the current layer are feature maps of all the previous layers, and its own feature maps are used as the input of all the subsequent layers, such as shown in Figure 4. Compared with a shortcut connection of the ResNet model, the special connection structure of DenseNet can effectively promote features reuse, which can realize better performance than ResNet in the case of less parameters and calculation costs. This connection structure can strengthen feature propagation, thereby alleviating the problem of vanishing gradients.

In DenseNet, the L layer output of the feature map $x_{l}$ can be described by Equation (1):

(1) $$x_{l}=H_{l}\left(Concat\left(x_{0},x_{1},\ldots ,x_{l-1}\right),\;W_{l}\right),$$

where $x_{0},x_{1},\ldots ,x_{l-1}$ are feature maps from layers 0, 1, …, l−1, respectively. Concat is a connection function that connects feature maps by channel dimensions. $H_{l}\left(\cdot \right)$ is a non-linear transformation function that represents some operations, including BN, ReLU, pooling, and convolution with the weights $W_{l}$.

#### 3.1.1. The Architecture of Mainstream Network

**Figure 5:**
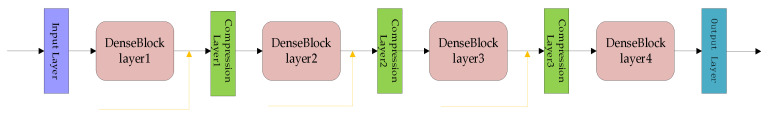
The architecture of the mainstream network.

The mainstream network mainly learns the similarity of dynamic deep features in gait images, that is, learning the changes in the stride, knee bending angle, arm swing amplitude, and body center of gravity during the walking process by DenseNet [19]. The architecture is shown in Figure 5.

The input of the network is a fixed-size 128 × 128 gait image (in order to better adapt to the network, we resize the 240 × 240 image to 128 × 128). The input layer contains one convolutional layer that applies a convolution kernel of 7 × 7 with a stride of 2 pixel and one max-pooling layer that applies 3 × 3 sliding windows with a stride of 1 pixels. The purpose of the input layer is to extract multi-scale basic visual features and reduce the image size to reduce network parameters. Each DenseBlock layer contains a DenseLayer (set to 2, 4, 8, 6 respectively). Each DenseLayer contains two convolution layers that apply a 1 × 1 kernel with a stride of 1 pixel and 3 × 3 kernel with a stride of 1 pixel—that is, BN-ReLU-Conv (1 × 1)-BN-ReLU-Conv (3 × 3). The compression layer uses a convolution kernel of 1 × 1 with a stride of 1 pixel and an avg-pooling kernel of 2 × 2 with a stride of 2 pixels to reduce the image to half its size. The purpose of the compression layer is to adjust the dimensions and further improve the compactness of the model. In this way, the feature map size of the auxiliary stream network input to the mainstream network can be reduced on the one hand, and the number of feature maps input to the next DenseBlock is reduced on the other hand. The output layer applies an average pooling layer with a sliding window of 8 × 8 and a 62-dimensional fully connected layer to obtain the final gait features. Finally, the similarity of gait images is obtained through the sigmoid function.

In the training task of gait recognition, because the network inputs a pair of gait images to learn their similarity, the training sample label is 1 (positive sample) or 0 (negative sample). A value of 1 means that two gait images are from same person, and a value of 0 means that two gait images are from different people. Therefore, our network uses a binary cross-entropy loss function to calculate the loss, as in Equation (2):

(2) $$loss=-\frac{1}{N}\left[\sum_{i=1}^{N}y_{i}\cdot \log\left(p\left(y_{i}\right)\right)+(1-y_{i})\cdot \log\left(1-p\left(y_{i}\right)\right)\right].$$

N represents the number of samples; $y_{i}$ represents the label value of sample i; and $p\left(y_{i}\right)$ represents the predicted probability value of the label value of sample i.

In the TS-Net model, the Adam [30] stochastic optimization algorithm is used to perform parameter updates. Adam is an efficient optimization algorithm, because the first moment estimation (mean of gradient) and second moment estimation (variance of gradient) are considered together, which makes the back-propagation algorithm easier to execute.

#### 3.1.2. Reducing Overfitting

In general, the deeper the model is during training, the more parameters need to be learned, making it easier to overfit. We applied the dropout [31,32] method before the last fully connected layer to solve this problem. Dropout means that during the training of the network, the input and output neurons are unchanged, and the hidden neurons are temporarily dropped from the network according to a certain probability—that is, the output is set to zero with a certain probability. The neurons that are “dropped out” participate in neither forward propagation nor backward propagation. Obviously, the network samples different architectures for each input, but these different architectures share identical weight. Therefore, dropout can effectively prevent overfitting of the training data. In this paper, we set dropout to 50% (usually 30% or 50%, empirically chosen in practical applications).

### 3.2. Auxiliary Stream Network

**Figure 6:**
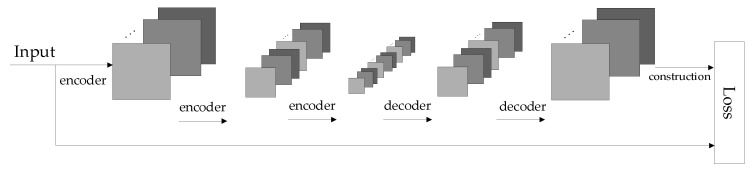
The architecture of auxiliary stream network.

After the mainstream network learns the similarity of dynamic deep features in the gait image, the auxiliary stream network is used to learn the similarity of static invariant features, including static information such as body shape, head shape, and shoulder width. The auxiliary stream network is designed based on a stacked convolutional autoencoder (SCAE). The architecture during the experiment is shown in Figure 6.

SCAE is composed of multiple convolutional autoencoders (CAE). CAE is a neural network designed to copy input to output. The network is divided into two parts: an encoder and decoder. The encoder compresses the input into a latent spatial representation, and the decoder is used to reconstruct this representation [33,34]. The purpose of CAE is to extract the most representative information to represent the original image—that is, the process of image dimensionality reduction. Compared with traditional dimensionality reduction methods such as PCA, the information extracted by the convolutional neural network is more effective and representative with the better recovery effect. The encoder network can be expressed by a neural network function passed by the activation function. The encoder network is defined as:

(3) z=σ (Wx+b)

where z denotes the hidden dimension of the encoder. W denotes the weight of the encoder network. b denotes the bias, and σ represents the non-linear activation function.

Similarly, the decoder network can be expressed in the same way. However, different weights, bias, and activation functions are applied, and it is defined as follows:

(4) $$x^{\prime }=\sigma \prime \left(W\prime z+b\prime \right).$$

Here, $x^{\prime }$ denotes the hidden dimension of the decoder.  W ′ denotes the weight of the decoder network. b′ denotes the bias, σ′ represents the non-linear activation function, and z represents the hidden dimension of the encoder.

The higher the similarity between the original data and the reconstructed data, the more effective the features extracted by the network. Therefore, we update the network by decreasing the discrepancy between input and output. The auxiliary stream network uses the mean squared error loss function to calculate the loss of the original gait image and the reconstructed gait image, which is expressed as:

(5) $$J\left(X,Y\right)=\frac{1}{2uv}\overset{u}{\underset{i=1}{\sum }}\overset{v}{\underset{j=1}{\sum }}{\left|\left|x_{ij}-y_{ij}\right|\right|}^{2}+\frac{\lambda }{2}{\left|\left|W\right|\right|}^{2}$$

where $x_{ij}$ and $y_{ij}$ denote the pixel values corresponding to the *i*-th row and the *j*-th column of the original gait image and the reconstructed gait image. u and v denote the number of rows and columns of the input data, respectively. The term $\frac{\lambda }{2}{\left|\left|W\right|\right|}^{2}$ is used for the weight decay.

In this paper, the auxiliary stream network is composed of three CAEs that have one hidden layer. During the training process, each CAE is individually trained. The output of the previous CAE is used as the input of the next CAE to achieve the purpose of “Each layer iterates; only a single layer updates”. In this way, the training revenue of the next CAE will be very high, because the input is the mapped features from the previous CAE training.

**Figure 7:**
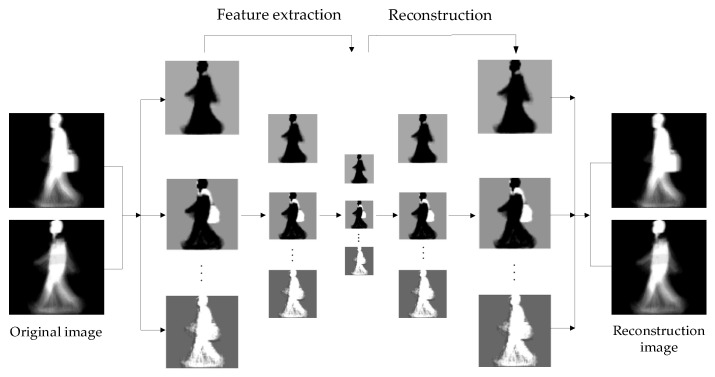
Reconstructed visualization process.

The auxiliary stream network extracts hierarchical features from gait image samples. During the feature extraction process, as the number of layers increases, the resolution of the feature map becomes smaller and smaller, and the blurriness gradually increases. However, the extracted static invariant features are increasingly obvious. The resemblance between the original image and the recovered image indicates that the extracted features retain the most significant information. The reconstructed visualization process is shown in Figure 7.

### 3.3. Feature Fusion and Recognition

**Figure 8:**
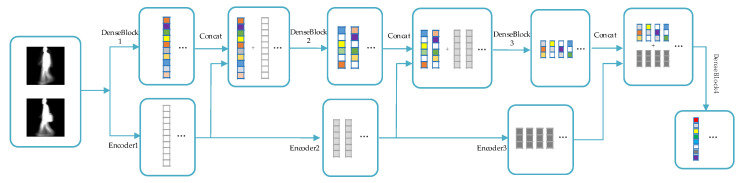
Process of the feature fusion method.

In this paper, a novel feature fusion method is used, as shown in Figure 8. We feed the multi-scale static invariant features extracted by the auxiliary stream network to the mainstream network, respectively. Compared with the traditional feature fusion method, which adds different features directly, our proposed method achieves feature reuse. As the depth of the model increases, the proportion of distinctive features becomes larger and larger, and the proportion of indistinguishable features becomes smaller and smaller. Therefore, the similarity of the image pair can be judged more accurately. The mainstream network fuses the features extracted by itself and the features extracted by the auxiliary stream network. The final gait feature features include both the dynamic and static features of human walking. Experiments show that this feature fusion method is very effective.

During the training task, the gait feature vector obtained by the mainstream network represents the similarity of a pair of gait images. We use the sigmoid function to convert the feature vector value to a value between 0 and 1. That is, the recognition task is transformed into a binary classification problem through similarity learning methods. If it is greater than 0.5, it is judged as a positive example—that is, the pair of gait images comes from the same person. Less than 0.5 is judged as a negative example—that is, the pair of gait images comes from different people. In the test task, we combine the probe view with each gallery view to form an image pair. Through the TS-Net model, we obtain the similarity of each pair of gait images. The person with the highest number of positive examples—that is, the person with the highest similarity—is the final recognition result. In order to better describe our model, the pseudocode for training and testing is shown in the Algorithm 1.
**Algorithm 1** TS-Net Model.Training: **Input data:**   image pair (X1, X2) randomly selected from the training set. **for**   i ← 1 to M (Iteration) **input**   SCAE ← (X1, X2) **output**   S (s1, s2, s3) ← SCAE **input**   DenseNet ← (X1, X2) and S(s1,s2, s3) **output**   Y ← DenseNet **do**   Loss ← $Loss(\theta_{s})$   ∆$\theta_{s}\leftarrow \frac{\partial Loss}{\partial \theta_{s}}$, ∆$\theta_{s}\;\leftarrow \;\theta_{s}$+$\alpha_{s}$∆$\theta_{s}$ (α is learning rate) **do**   Loss ← $Loss(\theta_{d})$   ∆$\theta_{d}\leftarrow \frac{\partial Loss}{\partial \theta_{d}}$, ∆$\theta_{d}\leftarrow \theta_{d}$+$\alpha_{d}$∆$\theta_{d}$ **end** Testing: **for**   $P_{i}\leftarrow P_{1}$ to $P_{n}$ (Images of prob set) **for**   $G_{i}\leftarrow G_{1}$ to $G_{n}$ (Images of gallery set) **input**   SCAE ← ($P_{i}$, $G_{i}$) **output**   S (s1, s2, s3) ← SCAE **input**   DenseNet ← ($P_{i}$, $G_{i}$) and S(s1,s2, s3) **output**   sim ← DenseNet   prediction ← vote(max(sim)) **end**

## 4. Experimental Results and Conclusions

### 4.1. Dataset

#### 4.1.1. CASIA-B Dataset

**Figure 9:**
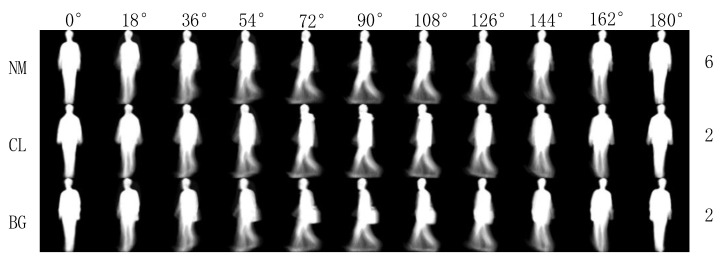
CASIA-B dataset.

First, we used the CASIA-B dataset [35], one of the largest public gait datasets created by the Institute of Automation, Chinese Academy of Sciences in 2005, to test the recognition performance of the proposed TS-Net model. The database contains 124 subjects (93 male and 31 female). The subject’s angle of view divided $0^{{}^{\circ}}$ to $180^{{}^{\circ}}$ into 11 different angles at $18^{{}^{\circ}}$ intervals. Each subject was divided into three walking states, including six normal walking sequences (NM), two walking with a bag sequences (BG), and walking with a coat sequences (CL), as shown in Figure 9.

#### 4.1.2. UCMP-GAIT Dataset

**Figure 10:**
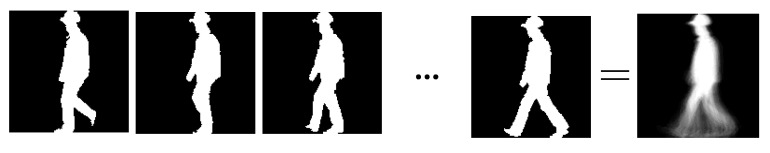
Gait energy image in collected gait dataset of underground coal mine personnel (UCMP-GAIT).

There is currently no public gait dataset for underground coal mine personnel (UCMP-GAIT). Therefore, in order to further verify the feasibility of the model for gait recognition of coal miners, we collected the gait data of 30 coal miners (all male, usually male workers under the mine). The UCMP-GAIT dataset is constructed as shown in Figure 10. The gait behavior of underground coal mine personnel is related to work content, environment, and dress. Therefore, the dataset contains 10 workers in each of the three types of work, namely coal miner, hydraulic support workers, and shearer driver. Each subject in the dataset contains 3 angles ($18^{{}^{\circ}}$, $54^{{}^{\circ}}$, $90^{{}^{\circ}}$). Each subject contains 2 walking sequences. One is taken in the coal mine examination room (with sufficient light and wide space), which is used as gallery views. The other is taken in the underground coal mine (dim light, limited space, wet, coal dust), which is used as probe views.

### 4.2. Experimental Design

In the experiments, the three walking states in the CASIA-B dataset, including “NM”, “BG”, and “CL” are involved. We used the six “NM” sequences, two “BG” sequences, and two “CL” sequences of the first 62 subjects (001–062) in the dataset as the training set. The remaining 62 subjects (063–124) were used as the test set. In the test set, the first 4 “NM” sequences of each subject are used as the gallery view, and the remaining two “NM” sequences, two “BG” sequences, and two “CL” sequences are used as the probe view to test the performance of the model in different walking states.

In the UCMP-GAIT dataset, all gait images of 30 coal miners were used to test the model. The gait image sequence captured in the coal mine examination room is used as the gallery view, and the sequence captured in the underground coal mine is used as the probe view.

### 4.3. Model Parameters

We set the batch size to 64. In addition, we use the Gaussian distribution with a mean of 0 and a standard deviation of 0.01 to initialize the weights of each layer. All biases are initialized to 0. In order to make the network converge better, we set the learning rate to 0.0001. We determine the number of iterations based on the recognition results on the validation set. The parameters are shown in Table 1.

#### 4.3.1. Mainstream Network Parameters

Too deep a network and too many feature maps will cause the model to be too complicated, require too many parameters, and take too long to identify. Too shallow a network and too few feature maps will cause the model to fail to learn discriminative features in the gait image and poor recognition results. Therefore, the optimal parameter settings obtained through multiple experiments are shown in Table 2. Each “conv” in the table corresponds to the BN-ReLU-Conv mode in the experiment.

#### 4.3.2. Auxiliary Stream Network Parameters

In the experiment, the auxiliary stream network parameters are divided into two parts: an encoder and decoder. The encoder is divided into 3 layers, and the decoding is also divided into 3 layers. The detailed parameters are shown in Table 3:

### 4.4. Experimental Results

Firstly, we conducted experiments in the CASIA-B test set (62 subjects). In order to evaluate the robustness of the TS-Net model, three variations covering view, clothing, and carrying objects are evaluated. There are 11 views in the database with a total of 121 pairs. We combined the probe view with the same angle view of all the subjects (62 subjects) in the gallery set (4 per subject) to composed into image pairs, and input into our proposed model to get the similarity (values between 0 and 1, closer to 1 indicates more similar). The experimental results are shown in Table 4, Table 5 and Table 6. Each row and each column in the table correspond to the angle of the gallery view and the probe view, respectively.

Secondly, in order to better verify the performance of the model, we interchange the testing set and the training set. We set the first 62 subjects (001–062) in the dataset as the testing set, and the remaining 62 subjects (063-124) were used as the training set. The experimental results are shown in Table 7. Experimental results show that our model performs stably and still achieves a higher recognition rate.

**Figure 11:**
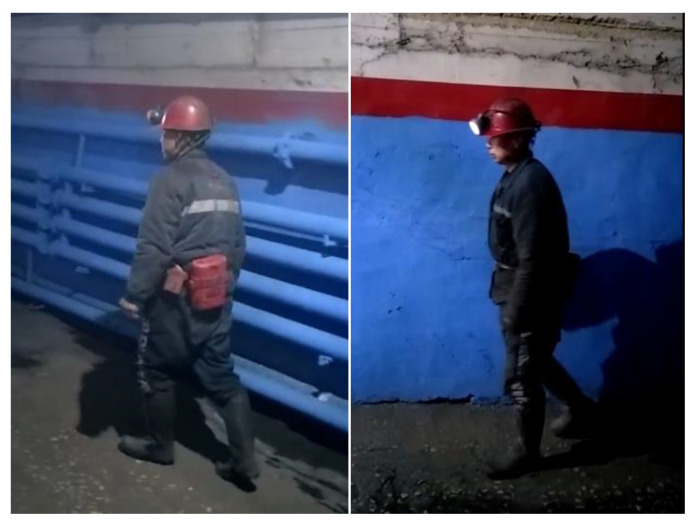
Gait images of underground coal mine personnel.

Finally, we conducted experiments on the UCMP-GAIT dataset, and the identity recognition rate was shown in Table 8. Gait recognition has little effect on environmental factors such as light and distance. Compared with the subjects in CASIA-B, the difference is that the personnel in the coal mines wear miner hats on their heads, carry tool bags on their bodies, and wear waterproof shoes on their feet, as shown in Figure 11. However, our model still has a high recognition rate, indicating that our proposed gait recognition method is also robust to these unique features of underground coal mine personnel.

### 4.5. Compared with State-of-the-Art Methods

We compare the proposed TS-Net model with the latest gait recognition method on the CASIA-B dataset, including deep convolutional neural networks (represented as CNNs) [12], principal component analysis (represented as GEI + PCA) [22], generate adversarial network (represented as GaitGAN) [13], and a perspective transformation model (represented as SPAE) [33].

**Figure 12:**
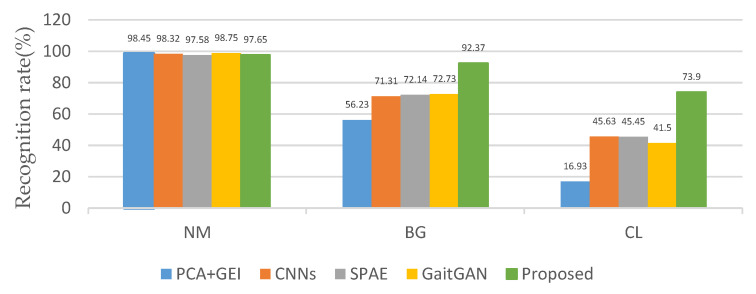
Recognition rate without perspective changes.

Firstly, we compared the recognition rate without angle changes—that is, the angle of the probe view and the gallery view are the same. The average recognition rate can be obtained by taking the recognition rates on the diagonals of Table 4, Table 5 and Table 6. The corresponding CNNs, GaitGAN, GEI + PCA, ResNet, and SCAE average rates are also obtained in the same way. The proposed method has a high recognition rate, as shown in Figure 12. Especially in the case of BG and CL, the recognition rates are 92.37% and 73.9%, which is significantly higher than other methods.

**Figure 13:**
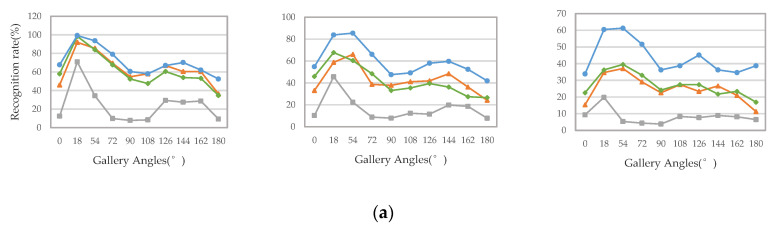

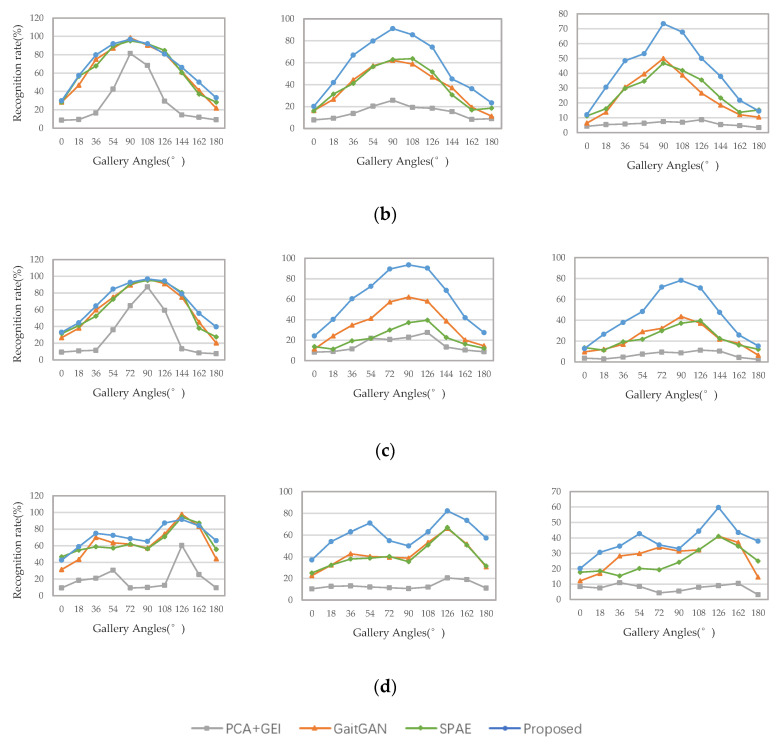
(**a**–**d**) represent the cross-view recognition rate of “NM”, “BG”, and “CL” walking condition when the probe view is $36^{{}^{\circ}}$, $72^{{}^{\circ}}$, $108^{{}^{\circ}}$, $144^{{}^{\circ}}$, respectively.

Secondly, we compared the recognition accuracy in the cross-view case—that is, the perspectives of the probe view and the gallery view are different. We select three walking conditions when the probe view is $36^{{}^{\circ}}$, $72^{{}^{\circ}}$, $108^{{}^{\circ}}$, $144^{{}^{\circ}}$, and the comparison results are shown in Figure 13. It can be seen from the results that our proposed method is significantly better than these methods, regardless of whether the viewing perspective changes.

Thirdly, we compared the overall recognition rate of the model with the SPAE [33] and MGAN [36] methods, as shown in Table 9. The results show that our model has a higher overall recognition rate. Especially in the presence of noise, the recognition effect is significantly higher than other methods.

Finally, we compared the recognition rates in UCMP-GAIT, as shown in Table 10. The gait recognition accuracy of the proposed TS-Net model increased by 6.67% compared with the recognition methods that have the highest accuracy.

The performance of our proposed TS-Net model is much better than the state-of-the-art gait recognition methods. No matter whether a cross-view or identical-view, the performance of the proposed model is demonstrated. The GEIs of BG, CL, and UCMP-GAIT contain massive but different noises, but the model still has a high recognition rate in these cases, indicating that our proposed model can eliminate the effects of noise to obtain the most discriminating features in GEIs. Our proposed model has good robustness to these noises; this is mainly due to the use of efficient multi-scale feature extraction and novel feature fusion techniques. At the same time, the TS-Net model based on DenseNet and SCAE has better performance, indicating that the multi-scale feature fusion of static invariant features and dynamic deep features are much better than a single static feature or dynamic feature. This is why our model has a high recognition rate.

### 4.6. Efficiency

Finally, we analyzed the computational cost of the model. In the calculation process, the main time-consuming step is in the mainstream network. In the testing process, the auxiliary stream network only needs to provide the encoded feature map, so there is no need to perform the most time-consuming decoding operation. The mainstream network needs to calculate the feature map generated by itself and the feature map from the auxiliary flow network. However, we have done a lot of optimization on the model, which greatly reduces the computational cost. Firstly, we use a convolutional layer in the input layer, which applies a convolution kernel of 7 × 7 with a stride of 2 pixe to reduce network parameters. Secondly, the compression layer will be used to reduce the size of the feature map to half after feeding the feature maps. In this way, the feature map size can be reduced on the one hand, and the number of feature maps input to the next DenseBlock is reduced on the other hand. Finally, the output layer applies a global pooling layer to reduce output parameters.

We ran the proposed method on a server with 4 Titan X (12 GB) GPU. For our experiment, only the inference time is measured on 1 GPU. After experimental calculation, the average time for inputting a gait image pair into our model to obtain the similarity is 2.73 ms. It takes a total of 22.11 s to predict 30 people on the UCMP-GAIT dataset. The average prediction time for a person is 0.25 s. Under the premise of ensuring the calculation efficiency, the accuracy of our model has been greatly improved. If you add batch processing, which can make sure the algorithm maximizes GPU capability or use a GPU with better computing performance, the computing efficiency of our models can be further improved.

## 5. Conclusions and Outlook

This paper proposes a TS-Net model based on DenseNet and SCAE, which is used to extract and fuse the dynamic deep features and static invariant features of gait images for the gait recognition of underground coal mine personnel. Mainstream networks use DenseNet to learn dynamic deep features to represent the macroscopic spatiotemporal characteristics of gait images. Auxiliary stream networks use SCAE to learn static invariant features, which are used to provide a low-dimensional physiology of gait image information. Then, the pixel-level dynamic deep features and hierarchical static invariant features are fused together to realize gait identification based on the similarity learning method. The proposed TS-Net model not only has a high recognition rate, but it also has good robustness to personnel angle changes, carrying conditions, miner hats, and clothing. The experimental results show that the proposed TS-Net model has a gait recognition accuracy of 92.22% in the UCMP-GAIT dataset, which is significantly better than the state-of-the-art gait recognition methods. What’s more, it is effective and feasible for underground coal mine personnel gait recognition.

In the underground coal mine, without being restricted by the complicated environment and by the distance, the gait recognition will play a vital role in identifying the personnel in the coal mine. The gait recognition of underground coal mine personnel needs to recognize the identity of the underground coal mine in real time, and the model recognition speed must be fast. Therefore, simplifying the model complexity and improving the recognition speed will become the focus of our future research work. In the future, we plan to collect more gait images of coal mine personnel to enrich our dataset, and study more in-depth models to improve the accuracy of gait recognition.

## Figures and Tables

**Table 1:** Training parameters.

Parameter	Optimization
Batch Size	64
Epochs	200,000
Learning Rate	0.0001

**Table 2:** Mainstream network parameters.

Layers	Output Size	Feature Num	Mainstream Neural Network
Convolution	64 × 64	2 → 24(12 × 2)	7 × 7 conv, stride 2
Pooling	64 × 64	24 → 24	3 × 3 max pool, stride 1
Dense Block (1)	64 × 64	24 → 48(24 + 12 × 2)	$\left[\begin{array}{c} 1\times 1\;\mathrm{conv}\\ 3\times 3\;\mathrm{conv} \end{array}\right]\;$ × 2
Compression Layer (1)	32 × 32	64(48 + 16) → 24	1 × 1 conv
			2 × 2 average pool, stride 2
Dense Block (2)	32 × 32	24 → 72(24 + 12 × 4)	$\left[\begin{array}{c} 1\times 1\;\mathrm{conv}\\ 3\times 3\;\mathrm{conv} \end{array}\right]\;$ × 4
Compression Layer (2)	16 × 16	104(72 + 32) → 36	1 × 1 conv
			2 × 2 average pool, stride 2
Dense Block (3)	16 × 16	36 → 132(36 + 12 × 8)	$\left[\begin{array}{c} 1\times 1\;\mathrm{conv}\\ 3\times 3\;\mathrm{conv} \end{array}\right]\;$ × 8
Compression Layer (3)	8 × 8	196(132 + 64) → 66	1 × 1 conv
			2 × 2 average pool, stride 2
Dense Block (4)	8 × 8	66 → 138(66 + 12 × 6)	$\left[\begin{array}{c} 1\times 1\;\mathrm{conv}\\ 3\times 3\;\mathrm{conv} \end{array}\right]\;$ × 6
Classification Layer	1 × 1	138 → 138	8 × 8 global average pool
			Fully connected, sigmoid

**Table 3:** Auxiliary stream network parameters.

Layers	Number of Filters	Filter Size	Stride	Batch Norm	Activation Function
Conv.1	16	2 × 2 × 2	2	Y	ReLU
Conv.2	32	2 × 2 × 16	2	Y	ReLU
Conv.3	64	2 × 2 × 32	2	Y	ReLU
F-Conv.1	64	2 × 2 × 32	1/2	Y	ReLU
F-Conv.2	32	2 × 2 × 16	1/2	Y	ReLU
F-Conv.3	16	2 × 2 × 2	1/2	Y	ReLU

**Table 4:** Multi-view recognition rate under normal walking condition.

Gallery view	Probe view (nm05, nm06)											
		0	18	36	54	72	90	108	126	144	162	180
	0	97.58	89.52	67.74	54.84	29.84	30.65	33.06	36.29	42.74	61.29	83.87
	18	87.10	98.39	99.19	87.90	57.26	45.16	44.35	54.84	58.7	64.52	65.32
	36	70.97	91.94	97.58	95.97	79.84	64.52	64.52	74.19	75.00	66.94	60.48
	54	46.77	74.19	93.55	96.77	91.94	80.65	84.68	82.26	72.58	54.03	38.71
	72	32.26	47.58	79.03	97.58	96.77	94.35	92.74	84.68	68.55	45.97	30.65
	90	28.23	37.10	60.48	85.48	96.77	97.58	96.77	89.52	65.32	41.94	28.23
	108	25.81	38.71	58.06	76.61	91.94	97.58	97.58	95.97	87.10	48.39	30.65
	126	33.06	51.61	66.94	76.61	80.65	87.10	94.35	96.77	91.13	74.19	46.77
	144	40.32	62.10	70.16	66.94	66.13	72.58	79.03	91.13	89.38	86.29	73.54
	162	57.26	70.97	62.10	54.03	50.00	45.97	55.65	75.00	83.87	99.19	87.10
	180	75.81	63.71	52.42	40.32	33.06	31.45	39.52	46.77	66.13	85.48	97.58

**Table 5:** Multi-view recognition rate under walking with a bag condition.

Gallery view	Probe view (bg01, bg02)											
		0	18	36	54	72	90	108	126	144	162	180
	0	91.94	75.00	54.84	35.48	20.16	16.13	24.19	27.42	37.10	56.45	62.90
	18	79.03	95.97	83.87	66.94	41.94	32.26	40.32	49.19	54.03	61.29	54.84
	36	52.42	82.26	90.32	87.90	66.94	49.19	60.48	70.16	62.90	48.39	42.74
	54	37.90	62.10	85.48	91.94	79.84	67.74	72.58	70.97	70.97	45.16	35.48
	72	20.97	32.26	66.13	91.13	98.39	93.55	89.52	77.42	54.84	41.13	25.00
	90	17.74	24.19	47.58	71.77	91.13	91.94	93.55	78.23	50.00	32.26	19.35
	108	22.58	31.45	49.19	71.77	85.48	88.71	93.55	91.94	62.90	37.90	25.00
	126	27.42	40.32	58.06	70.16	74.19	73.99	90.32	93.55	82.26	53.23	36.29
	144	33.87	52.42	59.68	58.87	45.16	48.39	68.55	87.10	88.71	81.45	48.39
	162	52.42	61.29	52.42	38.71	36.29	30.65	41.94	57.26	73.39	89.52	75.81
	180	59.67	53.23	41.94	30.65	23.39	16.13	27.42	32.26	57.26	84.68	90.32

**Table 6:** Multi-view recognition rate under walking in a coat condition.

Gallery view	Probe view (cl01, cl02)											
		0	18	36	54	72	90	108	126	144	162	180
	0	68.55	53.23	33.87	20.97	12.10	8.87	12.90	15.32	20.16	45.16	45.97
	18	46.77	67.74	60.48	41.13	30.65	28.23	26.61	31.34	30.65	34.68	29.03
	36	34.68	60.48	75.81	60.48	48.39	36.29	37.90	37.10	34.68	29.84	27.41
	54	21.77	47.58	61.29	75.00	53.23	50.00	48.39	41.94	42.74	27.42	25.00
	72	12.90	37.10	51.61	75.00	82.26	73.39	71.77	54.84	35.48	21.77	17.74
	90	17.74	26.61	36.29	54.03	73.39	75.81	78.23	62.90	33.06	20.16	15.32
	108	14.52	26.61	38.71	50.81	67.74	70.97	83.87	73.39	44.35	25.81	16.13
	126	19.35	35.48	45.16	49.19	50.00	55.65	70.97	78.23	59.68	40.32	27.42
	144	23.39	30.65	36.29	34.68	37.90	32.26	47.58	59.68	75.81	56.45	36.29
	162	38.71	50.00	34.68	21.77	21.77	22.58	25.81	33.87	43.55	63.71	47.58
	180	49.19	41.94	38.71	24.19	14.52	12.90	15.32	19.35	37.90	53.23	70.16

**Table 7:** Recognition rate after interchange the dataset.

Probe View		0°	18°	36°	54°	72°	90°	108°	126°	144°	162°	180°	Mean
Before swap	NM	54.10	65.95	73.39	75.73	70.38	67.96	71.11	75.22	73.61	66.20	58.45	68.37
	BG	45.09	55.57	62.68	65.03	60.26	55.33	63.86	66.86	63.12	57.41	46.92	58.38
	CL	31.60	43.40	46.63	46.11	44.72	42.45	47.21	46.18	41.64	38.05	32..55	41.88
After swap	NM	55.62	64.87	72.46	74.81	71.32	66.63	68.54	76.20	73.81	65.79	59.81	68.16
	BG	46.73	55.49	61.45	64.39	62.52	54.78	66.84	67.52	62.59	56.99	48.76	58.91
	CL	33.87	43.97	43.69	44.22	42.88	40.77	45.11	43.76	41.48	39.13	33.74	41.14

**Table 8:** Gait recognition rates for UCMP-GAIT test sets.

Type of Work	$18^{{}^{\circ}}$	$54^{{}^{\circ}}$	$90^{{}^{\circ}}$	Mean (%)
Coal miner	90.00	100.0	100.0	96.67
Hydraulic support worker	90.00	90.00	100.0	93.33
Shearer driver	80.00	90.00	90.00	86.67
All	86.67	93.33	96.67	92.22

**Table 9:** Comparison of overall recognition rate in CASIA-B.

Probe View		0°	18°	36°	54°	72°	90°	108°	126°	144°	162°	180°	Mean
NM# 5-6	SPAE	49.3	61.5	64.4	63.6	63.7	58.1	59.9	66.5	64.8	56.9	44.0	59.3
	MGAN	54.9	65.9	72.1	74.8	71.1	65.7	70.0	75.6	76.2	68.6	53.8	68.1
	Proposed	54.1	66.0	73.4	75.7	70.4	68.0	71.1	75.2	73.6	66.2	58.5	68.4
BG# 1-2	SPAE	29.8	37.7	39.2	40.5	43.8	37.5	43.0	42.7	36.3	30.6	28.5	37.2
	MGAN	48.5	58.5	59.7	58.0	53.7	49.8	54.0	61.3	59.5	55.9	43.1	54.7
	Proposed	45.1	55.6	62.7	65.0	60.3	55.3	63.9	66.9	63.1	57.4	46.9	58.4
CL# 1-2	SPAE	18.7	21.0	25.0	25.1	25.0	26.3	28.7	30.0	23.6	23.4	19.0	24.2
	MGAN	23.1	34.5	36.3	33.3	32.9	32.7	34.2	37.6	33.7	26.7	21.0	31.5
	Proposed	31.6	43.4	46.6	46.1	44.7	42.5	47.2	46.2	41.6	38.1	32.6	41.9

**Table 10:** Recognition accuracy comparison with other methods in the UCMP-GAIT dataset.

Methods	Accuracy (%)
GEI + PCA	31.11
CNNs	85.56
SPAE	83.33
GaitGAN	81.11
Proposed	92.22

## References

[1] Chai Y., Xia T., Han W. (2012). State-of-the-Art on Gait Recognition. Comput. Sci..

[2] Huang L., Xu Z., Wang L., Hu F. A novel gait contours segmentation algorithm. Proceedings of the International Conference on Computer, Mechatronics, Control and Electronic Engineering (CMCE).

[3] Zhang S., Zhang C., Huang W. (2013). Personnel identification in mine underground based on maximin discriminant projection. J. China Coal Soc..

[4] Kumar H.P.M., Nagendraswamy H.S. Gait recognition: An approach based on interval valued features. Proceedings of the International Conference on Computer Communication and Informatics (ICCCI).

[5] Sun J., Wang Y. (2018). View-invariant gait recognition based on kinect skeleton feature. Multimed. Tools Appl..

[6] Lishani A.O., Boubchir L., Khalifa E., Bouridane A. (2018). Human gait recognition using GEI-based local multi-scale feature descriptors. Multimed. Tools Appl..

[7] Zhao X.L., Zhang X.H. (2017). Gait Recognition Based on Dynamic and Static Feature Fusion. Nat. Sci. J. Xiangtan Univ..

[8] Xue Z., Ming D., Song W., Wan B., Jin S. (2010). Infrared gait recognition based on wavelet transform and support vector machine. Pattern Recognit..

[9] Tao D., Li X., Wu X., Wu X., Maybank S. (2007). General Tensor Discriminant Analysis and Gabor Features for Gait Recognition. IEEE Trans. Pattern Anal. Mach. Intell..

[10] He K., Zhang X., Ren S., Sun J. Deep residual learning for image recognition. Proceedings of the IEEE Conference on Computer Vision and Pattern Recognition, Fontainebleau Resort.

[11] Goodfellow I., Pouget-Abadie J., Mirza M., Xu B., Warde-Farley D., Ozair S., Courville A., Bengio Y. Generative adversarial nets. Proceedings of the Advances in Neural Information Processing Systems.

[12] Wu Z., Huang Y., Wang L., Wang X., Tan T. (2017). A comprehensive study on cross-view gait based human identification with deep cnns. IEEE Trans. Pattern Anal. Mach. Intell..

[13] Yu S., Chen H., Reyes E.B.G., Poh N. GaitGAN: Invariant Gait Feature Extraction Using Generative Adversarial Networks. Proceedings of the 2017 IEEE Conference Computer Vision and Pattern Recognition (CVPR).

[14] Chao H., He Y., Zhang J., Feng J. (2018). GaitSet: Regarding Gait as a Set for Cross-View Gait Recognition. arXiv.

[15] Wu L., Cheng Z. (2018). Learning Efficient Spatial-Temporal Gait Features with Deep Learning for Human Identification. Neuroinformatics.

[16] Zhang Y., Huang Y., Wang L., Yu S. (2019). A comprehensive study on gait biometrics using a joint CNN-based method. Pattern Recognit..

[17] Wang X., Zhang J. (2020). Gait feature extraction and gait classification using two-branch CNN. Multimed. Tools Appl..

[18] Mehmood A., Khan M.A. (2020). Prosperous Human Gait Recognition: An end-to-end system based on pre-trained CNN features selection. Multimed. Tools Appl..

[19] Huang G., Liu Z., Weinberger K.Q., van der Maaten L. Densely connected convolutional networks. Proceedings of the IEEE Conference on Computer Vision and Pattern Recognition.

[20] Tao Y., Xu M., Zhong Y., Cheng Y. (2017). GAN-Assisted Two-Stream Neural Network for High-Resolution Remote Sensing Image Classification. Remote Sens..

[21] Hu J., Mou L., Schmitt A., Zhu X.X. FusioNet: A Two-Stream convolutional neural network for urban scene classification using PolSAR and hyperspectral data. Proceedings of the Urban Remote Sensing Event (JURSE).

[22] Han J., Bhanu B. (2006). Individual recognition using gait energy image. Trans. Pattern Anal. Mach. Intell..

[23] Vincent P., Larochelle H., Bengio Y., Manzagol P.A. Extracting and composing robust features with denoising autoencoders. Proceedings of the ACM 25th International Conference on Machine Learning.

[24] Masci J., Meier U., Cire¸san D., Schmidhuber J. Stacked convolutional auto-encoders for hierarchical feature extraction. Proceedings of the 21st International Conference on Artificial Neural Networks—Volume Part II.

[25] Glorot X., Bengio Y. (2010). Understanding the difficulty of training deep feedforward neural networks. J. Mach. Learn. Res..

[26] He K., Zhang X., Ren S., Sun J. Delving deep into rectifiers: Surpassing human-level performance on imagenet classification. Proceedings of the IEEE International Conference on Computer Vision (ICCV).

[27] Liu Y., Liu Y., Ding L. (2018). Scene Classification Based on Two-Stage Deep Feature Fusion. IEEE Geosci. Remote Sens. Lett..

[28] Yu Y., Gong Z., Wang C., Zhong P. (2018). An Unsupervised Convolutional Feature Fusion Network for Deep Representation of Remote Sensing Images. IEEE Geosci. Remote Sens. Lett..

[29] Song W., Li S., Fang L., Lu T. (2018). Hyperspectral Image Classification with Deep Feature Fusion Network. IEEE Trans. Geosci. Remote Sens..

[30] Kingma D.P., Ba J. (2014). Adam: A method for stochastic optimization. arXiv.

[31] Sokolova M., Guy L. (2009). A systematic analysis of performance measures for classification tasks. Inf. Process. Manag..

[32] Powers D.M.W. (2011). Evaluation: From Precision, Recall and F-Factor to ROC, Informedness, Markedness and Correlation. J. Mach. Learn. Technol..

[33] Yu S., Chen H., Wang Q., Shen L., Huang Y. (2017). Invariant feature extraction for gait recognition using only one uniform model. Neurocomputing.

[34] Yoo D., Kim N., Park S., Paek A.S., Kweon I.S. (2016). Pixel-level domain transfer. arXiv.

[35] Yu S., Tan D., Tan T. A framework for evaluating the effect of view angle, clothing and carrying condition on gait recognition. Proceedings of the IEEE of 18th International Conference on Pattern Recognition (ICPR).

[36] He Y., Zhang J., Shan H., Wang L. (2019). Multi-task GANs for view-specific feature learning in gait recognition. IEEE TIFS.

